# Genomic evidence of neo-sex chromosomes in the eastern yellow robin

**DOI:** 10.1093/gigascience/giz131

**Published:** 2019-12-30

**Authors:** Han Ming Gan, Stephanie Falk, Hernan E Moraleś, Christopher M Austin, Paul Sunnucks, Alexandra Pavlova

**Affiliations:** 1 Centre for Integrative Ecology, School of Life and Environmental Sciences, Deakin University, Geelong, Victoria 3220, Australia; 2 Deakin Genomics Centre, Deakin University, Geelong, Victoria 3220, Australia; 3 School of Biological Sciences, Monash University, Clayton Campus, Clayton, Victoria 3800, Australia; 4 Centre for Marine Evolutionary Biology, Department of Marine Sciences, University of Gothenburg, Go¨teborg 405 30, Sweden

In the final publication of “Genomic evidence of neo-sex chromosomes in the Eastern Yellow Robin,” by Gan et al., supplementary data files were incorrectly linked from another article. The correct supplementary data files for this article now appear online .

The publisher regrets this error.

