# Supplementary material for: Genomic evidence of neo-sex chromosomes in the eastern yellow robin
Source: Gigascience. 2019 Sep 3;8(9):giz111. doi: 10.1093/gigascience/giz111 (PMC6736294; doi:10.1093/gigascience/giz111)
Supplement: giz111_GIGA-D-19-00237_Original_Submission [file giz111_giga-d-19-00237_original_submission.pdf]

GigaScience

Genomic evidence of neo-sex chromosomes in the Eastern Yellow Robin

--Manuscript Draft--

**Manuscript Number:**	GIGA-D-19-00237
**Full Title:**	Genomic evidence of neo-sex chromosomes in the Eastern Yellow Robin
**Article Type:**	Data Note
**Funding Information:**	Australian Research Council (ARC) Discovery Project (DP180102359) — Prof Paul Sunnucks<br>ARC Linkage Grant (LP0776322) — Prof Paul Sunnucks

Abstract:

Background: Understanding sex-biased natural selection can be greatly enhanced by access to well-annotated chromosomes including ones inherited in sex-specific fashions. The Eastern Yellow Robin (EYR) is an endemic Australian songbird inferred to have experienced climate-driven sex-biased selection and is a prominent model for studying mitochondrial-nuclear interactions in the wild. However, the lack of an EYR reference genome containing both sex chromosomes (in birds, a female bearing Z and W chromosomes) is limiting current efforts to understand the mechanisms of these processes. Here, we assemble the genome for a female EYR and use low depth (10 ×) genome resequencing data from 19 individuals of known sex to identify chromosome fragments with sex-specific inheritance.

Findings: MaSurCA hybrid assembly using Nanopore and Illumina reads generated a 1.22 Gb EYR genome in 20,702 scaffolds (94.2% BUSCO completeness). Scaffolds were tested for W-linked (female-only) inheritance using a k-mer approach, and for Z-linked inheritance using median read-depth test in male and female reads (read-depths must indicate haploid female and diploid male representation). This resulted in 2,372 W-linked scaffolds (total length: 97,872,282 bp, N50: 81,931 bp) and 586 Z-linked scaffolds (total length: 121,817,358 bp, N50: 551,641 bp). Anchoring of the sex-linked EYR scaffolds to the reference genome of a female Zebra Finch revealed two categories of sex-linked genome region. First, 653 W-linked scaffolds (25.7 Mb) were anchored to the W sex chromosome and 215 Z-linked scaffolds (74.4 Mb) to the Z. Second, 1138 W-linked scaffolds (70.9 Mb), and 179 Z-linked scaffolds (51.0 Mb), were anchored to a large section (coordinates ~5 to ~60 Mb) of Zebra Finch chromosome 1A. The first ~5 Mb and last ~14 Mb of the reference chromosome 1A had only autosomally-behaving EYR scaffolds mapping to them.

Conclusions: We report a female (W-chromosome containing) EYR genome and provide genomic evidence for a neo-sex (neo-W and neo-Z) chromosome system in EYR, involving most of a large chromosome (1A) previously only reported to be autosomal in passerines.

**Corresponding Author:**	Han Ming Gan<br><br>AUSTRALIA
**Corresponding Author Secondary Information:**	
**Corresponding Author's Institution:**	
**Corresponding Author's Secondary Institution:**	
**First Author:**	Han Ming Gan
**First Author Secondary Information:**	
**Order of Authors:**	Han Ming Gan
	Stephanie Falk
	Hernán E. Morales

Powered by Editorial Manager® and ProduXion Manager® from Aries Systems Corporation

	Christopher M. Austin
	Paul Sunnucks
	Alexandra Pavlova
**Order of Authors Secondary Information:**	
**Additional Information:**	
**Question**	**Response**
Are you submitting this manuscript to a special series or article collection?	No
**Experimental design and statistics**<br><br>Full details of the experimental design and statistical methods used should be given in the Methods section, as detailed in our Minimum Standards Reporting Checklist. Information essential to interpreting the data presented should be made available in the figure legends.<br><br>Have you included all the information requested in your manuscript?	Yes
**Resources**<br><br>A description of all resources used, including antibodies, cell lines, animals and software tools, with enough information to allow them to be uniquely identified, should be included in the Methods section. Authors are strongly encouraged to cite Research Resource Identifiers (RRIDs) for antibodies, model organisms and tools, where possible.<br><br>Have you included the information requested as detailed in our Minimum Standards Reporting Checklist?	Yes
**Availability of data and materials**<br><br>All datasets and code on which the conclusions of the paper rely must be either included in your submission or deposited in publicly available repositories (where available and ethically	Yes

appropriate), referencing such data using a unique identifier in the references and in the “Availability of Data and Materials” section of your manuscript.<br><br>Have you have met the above requirement as detailed in our Minimum Standards Reporting Checklist?	

Powered by Editorial Manager® and ProduXion Manager® from Aries Systems Corporation

Manuscript

Click here to download Manuscript EYR_GigaScience_250619.docx

Genomic evidence of neo-sex chromosomes in the Eastern Yellow Robin

Han Ming Gan[1,2]*, Stephanie Falk[3], Hernán E. Morales[4], Christopher M. Austin[1,2], Paul Sunnucks[3], Alexandra Pavlova[3*]

[1] Centre for Integrative Ecology, School of Life and Environmental Sciences, Deakin University, Geelong, Victoria, Australia
[2] Deakin Genomics Centre, Deakin University, Geelong, Victoria, Australia
[3] School of Biological Sciences, Monash University, Clayton Campus, Clayton, Victoria, Australia
[4] Centre for Marine Evolutionary Biology, Department of Marine Sciences, University of Gothenburg, Göteborg, Sweden

*** Corresponding author:**

Name: Han Ming Gan, PhD
Address: Building KA4, School of Life and Environmental Sciences, Deakin University, Waurn Ponds, Victoria 3220, Australia
Phone: 0490786277
Email: han.gan@deakin.edu.au

Name: Alexandra Pavlova
Address: School of Biological Sciences
Monash University, Clayton Campus
Clayton Victoria 3800
Phone: 0399055902
Email: alexandra.pavlova@monash.edu

Abstract

Background: Understanding sex-biased natural selection can be greatly enhanced by access to well-annotated chromosomes including ones inherited in sex-specific fashions. The Eastern Yellow Robin (EYR) is an endemic Australian songbird inferred to have experienced climate-driven sex-biased selection and is a prominent model for studying mitochondrial-nuclear interactions in the wild. However, the lack of an EYR reference genome containing both sex chromosomes (in birds, a female bearing Z and W chromosomes) is limiting current efforts to understand the mechanisms of these processes. Here, we assemble the genome for a female EYR and use low depth (10 ×) genome resequencing data from 19 individuals of known sex to identify chromosome fragments with sex-specific inheritance.

Findings: MaSurCA hybrid assembly using Nanopore and Illumina reads generated a 1.22 Gb EYR genome in 20,702 scaffolds (94.2% BUSCO completeness). Scaffolds were tested for W-linked (female-only) inheritance using a *k*-mer approach, and for Z-linked inheritance using median read-depth test in male and female reads (read-depths must indicate haploid female and diploid male representation). This resulted in 2,372 W-linked scaffolds (total length: 97,872,282 bp, N_{50}: 81,931 bp) and 586 Z-linked scaffolds (total length: 121,817,358 bp, N_{50}: 551,641 bp). Anchoring of the sex-linked EYR scaffolds to the reference genome of a female Zebra Finch revealed two categories of sex-linked genome region. First, 653 W-linked scaffolds (25.7 Mb) were anchored to the W sex chromosome and 215 Z-linked scaffolds (74.4 Mb) to the Z. Second, 1138 W-linked scaffolds (70.9 Mb), and 179 Z-linked scaffolds (51.0 Mb), were anchored to a large section (coordinates ~5 to ~60 Mb) of Zebra Finch chromosome 1A. The first ~5 Mb and last ~14 Mb of the reference chromosome 1A had only autosomally-behaving EYR scaffolds mapping to them.

Conclusions: We report a female (W-chromosome containing) EYR genome and provide genomic evidence for a neo-sex (neo-W and neo-Z) chromosome system in EYR, involving most of a large chromosome (1A) previously only reported to be autosomal in passerines.

Keywords: Eastern Yellow Robin, *Eopsaltria australis*, passerine, songbird, genome, sex chromosome, W-chromosome, neo-W, neo-Z

Data description

Wildlife species that have genomic variation distributed heterogeneously through environmental and geographic space can be excellent models for studying evolutionary processes under natural conditions. Eastern Yellow Robin (EYR), *Eopsaltria australis,* is a common endemic eastern Australian songbird (Figure 1) that shows geographically discordant patterns of mitochondrial and nuclear genome variation. Whereas nuclear DNA variation in EYR is structured mainly north-to-south, its two mitochondrial lineages (mitolineages) occur in contrasting climates in an east-west (coast-to-inland) direction, with a narrow contact zone between them, despite ongoing male-mediated gene flow [1] . This pattern is inferred to have arisen when EYR experienced two instances of climate-driven mitochondrial introgression into different nuclear backgrounds: from the northern population into the southern through the inland, and from the southern into the northern population along the coast [2]. Because mitogenome divergence is mirrored by a fraction of the EYR nuclear genome that maps to the chromosome 1A of Zebra Finch and is enriched for genes with mitochondrial functions, each inferred mitochondrial introgression is hypothesized to have been accompanied by co-introgression of a co-evolved nuclear region [3]. Accordingly, the species has been highlighted as an exceptional model in the emerging field of 'mitonuclear ecology', which addresses evolutionary interactions between mitochondrial and nuclear genomes and their products [4].

Whereas progress on understanding mitonuclear interactions in EYR has been made by mapping genomic reads to a male Zebra Finch *Taeniopygia guttata* reference genome [5], the ~40 million years of evolution between the two species limits the assumptions that can be made about the degree of synteny of their genome organization. Moreover, the male reference lacks the female-specific W chromosome in birds. Nuclear genomic architecture (for example, concentrations of genes with mitochondrial functions that are subject to suppressed recombination), has considerable potential to be a driver of mitonuclear evolution [6]. Furthermore, female-specific selection has been inferred for EYR, based on fine-scale spatial separation of mitolineage distributions and their correlation with climate, despite male-biased gene flow in a species with female-biased dispersal [1]. Accordingly, genomic architecture with the potential to impact the sexes differently could be a key player in mitonuclear evolution in this species. Thus to test among alternative hypotheses concerning mechanisms of potential co-

evolution between elements of the nuclear genome and maternally-transmitted mtDNA, reference sequences of both sex chromosomes are required. For example, the female-specific W-chromosome is necessarily co-inherited with mitochondrial DNA, and a species could experience evolution so that W-chromosome bore genes relevant to mitochondrial function [1]. Substantial female-specific gene regions are known from birds, notably neo-sex chromosome systems that can provide females with gene sequences unavailable to males [7, 8] .

Using a combination of Illumina and Nanopore reads, which have been shown to produce contiguous genome assemblies [9-12], we assembled a female inland EYR reference genome and utilized population genomic data from populations harbouring only inland mitochondrial lineages [13] to identify and annotate W and Z sex chromosomes. This procedure could also detect sex-linked chromosomes other than the typical W and Z avian sex chromosomes such as neo-sex chromosomes (caused by fusions between autosomal and sex chromosome elements) that are uncommon but known in birds, notably throughout the Sylvioidea, and in a honeyeater [7, 8, 14-16]

Sample collection, library construction and sequencing

Two EYR females, EYR054 and EYR056, were captured at Stuart Mill, western Victoria in the same net on 6th of April 2009, as part of another project [17, 18]. DNA was extracted from 40 µL of blood using a Qiagen DNAeasy Blood and Tissue Kit. A standard paired-end Illumina library was constructed from 100 ng of QSonica-fragmented (~ 350 bp fragment size) EYR054 DNA using the NEBUltra Illumina Library Preparation kit (New England Biolabs, Ipwich, MA). The library was quantified with a Tapestation 4000 (Agilent) and sequenced on the Novaseq6000 (2 × 150 bp run configuration) at the Deakin Genomics Centre. Two Oxford Nanopore sequencing libraries were constructed from G-tube fragmented (~8 kb) EYR054 gDNA using the LSK108 library preparation kit. Sequencing was performed on two MinION R9.4.1 flowcells for 48 hours followed by fast5 base-calling using Albacore. A total of 6.63 Gb Nanopore data in 916,218 reads (N_{50} = 10,224 bp) were generated after adapter-trimming using Porechop v0.2.3 (https://github.com/rrwick/Porechop). Nanopore reads used for this study had 13% error rate, estimated based on mean pairwise sequence similarity of 87% (median= 89%) between Nanopore reads and the assembled EYR genome, aligned using Minimap2 [19]. The DNA of EYR056 was used to construct a mate-pair library with an insert size of 1 kb and sequenced by BGI for earlier studies [18]. EYR054 is similar genetically to EYR056 according to whole mitogenomes, microsatellites, and being female contemporaries in an area of the species' range where only the

inland mitolineage occurs, in an isolated habitat patch characterized by high local genetic relatedness [3, 18, 20].

For low (~10 ×) depth whole genome resequencing, 10 female and 9 male EYR individuals bearing inland mitogenomes (EYR-A) were selected from northern (N=9) and southern (N=10) populations [2, 13] away from the contact zone between the inland and coastal mitolineages. Prior to Illumina sequencing, EYR individuals were genetically sexed based on the intron length-variation of homologous sections of CHD (chromo-helicase-DNA-binding) genes located on W (female-limited) and Z (occurs in both sexes) chromosomes [21]. These fragments have been sequenced previously for EYR for both sexes [1]. DNA extraction from 16 blood samples and five tissues (Supplemental Table 1) was performed using a Qiagen DNAeasy extraction kit. Illumina library construction and whole genome sequencing were performed at the Deakin Genomics Centre using the methods described above, generating an average of 17 Gb (min = 12 Gb; max = 31 Gb) sequencing output per sample (Supplemental Table 1).

Genome size estimation, hybrid *de novo* assembly and annotation

Raw Illumina EYR054 reads were poly-G, adapter- and quality trimmed using fastp v0.18.0 [22]. The trimmed reads were used for genome profiling based on Jellyfish2-calculated *k*-mer frequency (k=25) that estimated a genome size of 993 Mb with 1.12% heterozygosity for EYR054 (Figure 2A) [22-24]. We used MaSuRCA v3.2.4 [25] to perform a hybrid assembly of the EYR054 Nanopore and poly-G trimmed Illumina reads followed by gap-closing with Sealer v2.0.2 [26]. For the MaSuRCA assembly, Illumina reads were first error-corrected and used to construct contigs using the de Bruijn graph approach. These contigs were then used to error-correct the Nanopore long reads generating "mega reads" contigs and used for Overlap-Layout-Consensus assembly. Subsequently, the MaSuRCA hybrid assembly was gap-closed with Sealer v2.0.2 using Illumina paired-end reads from the same individual. Given that EYR056 and EYR054 are from the same population away from the hybrid zone (Harrisson et al. 2012, Morales et al. 2018) and thus likely possess similar versions of chromosomes, the EYR054 assembly was further scaffolded with mate-pair data from EYR056 using BESST [27] to generate the final assembly for subsequent analyses (Table 1). Using mate-pair data improved the assembly N_{50} from 585 kb to 987 kb. The Sealer-gap-closed EYR054-only assembly is also made available in the GigaDB , should the future work on this species require single-individual assembly.

BUSCOv3 [28] assessment of the assembled genome based on the avian protein database (aves_odb9), indicates 94.2% genome completeness with a low level of duplicated genes (Table

1). Prior to gene prediction, the genome was masked for repeats using RepeatModeler v1.0.11 and RepeatMasker v4.0.7 [29, 30]. The soft-masked genome (15.77% masked, Table 1) along with the reference proteome of a male Collared Flycatcher [31] were used as the input for BRAKER2 annotation [32], resulting in the prediction of 23,905 genes. The Collared Flycatcher proteome was used here in preference to Zebra Finch because the former has greater protein similarity to EYR.

Identification of sex chromosome scaffolds

Scaffolds inherited in sex-specific fashions ('sex-linked', 'W-linked' or 'Z-linked') were identified using two methods (explained below) applied to sequence data obtained from 10 female and 9 male EYR individuals as detailed above. Paired-end reads for each re-sequenced male and female were poly-G, quality- and adapter-trimmed using fastp (default setting) [22]. The trimmed reads were mapped to the EYR genome using Bowtie2 v2.3.4 [33]. High mapping rates ranging from 97.82 to 98.53% were observed across all 19 individuals, indicating robust assembly of the female EYR genome. The read mapping quality reported by Bowtie2 is relatively constant (MapQ >30) across the assembly albeit with lower quality in the repetitive regions as short reads will not be able to uniquely map to these regions. Subsequently, 90 million mapped PE reads were subsampled from each individual (to equalize coverage across individuals) and used to estimate for each individual the median read-depth for each scaffold, and the fraction of the length of each scaffold that was covered by reads, using BAMStat04 as implemented in the jvarkit package [34, 35].

Genome-wide identification of sex-linked scaffolds based on pooled male and female reads could be compromised if any individuals had their sexes mis-assigned. Accordingly, to confirm the sex of the individual to which each set of sequence data was ascribed, the read-depth profiles for all 19 EYR were assessed for the CHD sexing region noted above. BLASTN was used to align the CHD-W and CHD-Z nucleotide sequences (GenBank accession KC466840 - KC466844 CHD-W and KC466845 - KC466853 CHD-Z) to two separate, long scaffolds (W chromosome scaffold: QKXG01001703.1 - 310,213 bp; Z chromosome scaffold: QKXG01001459.1 - 211,357 bp). For comparison, an autosomal scaffold, QKXG01002030.1 (3,864,097 bp) was identified that contained a fragment of the single-copy autosomal GAPDH (glyceraldehyde-3-phosphate dehydrogenase) gene, sequenced previously for EYR (Genbank accession KC466694- KC466739) [1]. For the Z chromosome scaffold, a median read-depth centered on ~5× (haploid depth) was observed in females, and ~10× (diploid) in males, while for the W chromosome fragment it was ~5× (haploid) in females and ~0× (absent) in males; ~10× diploid depth was observed for the

autosomal scaffold in both sexes (Figure 2B).

BAM files from individual EYR were merged by sex using samtools v1.9 [36] to generate one pooled alignment BAM file per sex. A histogram of read-depth frequency for each sex was then generated using ‘samtools depth’ to estimate the read-depth cut-off for the identification of candidate W- and Z-linked scaffolds (Figure 2C). The expected diploid depth for each sex was estimated based on the peak observed read-depth (male = 77×; female = 83×, Figure 2C). A minor peak corresponding to haploid read-depth (~40×) was observed for females but not males, consistent with females being hemizygous for sex-linked regions (Figure 2C). A strong peak of low read-depth sequences (<5x) was seen only for males, consistent with their lacking a W chromosome (Figure 2C).

To identify candidate W-linked scaffolds, we applied two established approaches with complementary strengths that take advantage of sequence data being available for each sex. First, we used a differential mapping approach, based on the expectation that a W-linked scaffold should exhibit zero median read-depth in males, with a more than 75% of the scaffold having female reads mapping to it [7, 8]. Second, we used the YGS (‘Y chromosome Genome Scan’) *k*-mer approach, designed for detecting W- or Y-linked regions [37]. The *k*-mer approach removes identical repetitive sequences that might lead to false-positive matches to W-linked regions while retaining useful information from unique variants of repetitive regions: this is an advantageous attribute in the face of the elevated repetitiveness expected of W chromosome sequences [37]. The *k*-mer approach was implemented as follows. For the pooled male reads, pooled female reads and the female EYR genome assembly dataset, separate lists were built of all overlapping 16-bp sequences (‘16-mers’): k=16 was chosen on the basis of genome size, and empirical validation that it produced bimodal frequency distributions of *k*-mer presences in larger (>1 Gb) genomes [37]. Then, scaffolds from the assembled female genome are assumed to be W-linked if >75% of their single-copy *k*-mers are absent in the pooled male reads but present in both of the female genome and pooled female reads.

Together, the two approaches identified 2,372 candidate W-linked scaffolds (total length of 97.87 Mb) that were used for downstream analyses. A great majority (1,952, 82.3 %, amounting to 86.32 Mb) of the candidate W-linked scaffolds were identified by both approaches, with 174 (7.3 %, 2.64 Mb) being exclusive to the *k*-mer approach, and 246 (10.4 %, 8.91 Mb) found only by the differential mapping approach. Inspection of the repetitiveness in the candidate W-linked scaffolds identified only by the *k*-mer approach indicates that they are 80% repetitive (total repeat length/total sequence length × 100%), consistent with the high sensitivity of *k*-mer approach in identifying repetitive sex-linked scaffolds [37]. In contrast, the candidate W-linked scaffolds found

by the differential mapping approach alone were only 32.6% repetitive.

Since Z-linked scaffolds are present in males and females, it is not possible to utilize the YGS *k*-mer approach to identify candidates. Thus we identified putative Z-linked scaffolds on the basis of differences in read-depth between males and females, similar to the differential mapping method for W-linked scaffold discovery outlined above. To allow for variation in sequencing depth, we conservatively defined a candidate Z-linked scaffold as one exhibiting more than 58× median read-depth in males (i.e. 0.75 times the observed male diploid read-depth of 77×) and less than 62× median read-depth in females (i.e. 1.5 times the observed female haploid read-depth of 41.5×). Scaffolds passing these thresholds were further filtered to retain only those having both male and female reads mapping to > 75% of the scaffold length. This resulted in the identification of 586 candidate Z-linked scaffolds with a total length of 121.8 Mb and N_{50} of 551.6 kb.

The total lengths of W-linked scaffolds (97.87 Mb) and Z-linked scaffolds = 121.82 Mb are much greater than expected from the typical sizes of sex chromosomes in Passerida, of which EYR is a member (e.g. in Xu *et al.* 2019 [38], Passerida W chromosomes range from 3.37-4.75 Mb and Z chromosomes range from 68.8-74.7 Mb) [38]. These observations raised the possibility of the presence of a neo-sex chromosome system, and hence it was of great interest to compare the sex-linked regions to a well-annotated reference genome, as follows.

Genomic evidence of neo-sex chromosomes in Eastern Yellow Robin

To assess the accuracy of our approaches for detecting sex-linked regions known in related reference genomes, and to test for possible neo-sex chromosomes, the candidate W- and Z-linked scaffolds were separately anchored to the female Zebra Finch genome (bTaeGut2: https://vgp.github.io/genomeark/Taeniopygia_guttata/, accessed on 19th December 2018) using RaGoo v1.0 (with default settings) [39]. A total of 215 Z-linked scaffolds (74.4 Mb,) were anchored to the Zebra Finch Z chromosome, and 653 W-linked scaffolds (25.7 Mb) to the Zebra Finch W chromosome. Surprisingly,A substantial proportion of candidate W-linked (n=1138, 70.9 Mb) and Z-linked (n=179, 51.0 Mb) scaffolds were also anchored to the autosomal Zebra Finch chromosome 1A. Thus, each sex-linked scaffold anchored to one of three female Zebra Finch chromosomes: W, Z or chromosome 1A. Using the entire EYR draft genome assembly as the RaGoo input led to the anchoring of several W- and Z-linked scaffolds with the best hits to the Zebra finch chromosome 1A, resulting in a substantially larger pseudomolecule for chromosome 1A (143.6 Mb), a length that is nearly double that of the Zebra Finch chromosome 1A (71.64 Mb) which suggests the presence of two separate sex-linked versions of chromosome 1A in EYR (Fig.

3). By re-anchoring the EYR scaffolds in the absence of first the candidate W-linked and then the candidate Z-linked scaffolds, two distinct versions of a chromosome 1A pseudomolecule were recovered that we designated putative neoZ-1A and neoW-1A chromosomes and used for subsequent analyses.

To assess the robustness of the sex-based scaffold assignment approach, and to check the sex-specific read-depth and length coverage along the putative neo-sex chromosomes involving chromosome 1A (which we refer to as "pseudomolecules neoW-1A (Fig. 3 Chr1A: pink bar) and neoZ-1A (Fig. 3 Chr1A: light blue bar)"), pooled female and male reads were mapped to the constructed EYR Z, W, autosomal chromosome 5, and neoZ-1A and neoW-1A pseudomolecules. The mean read-depth in 100 kb non-overlapping sliding windows was calculated using the 'coverage' command in bedtool v2.25.0 [40] and visualized with ggplot2 in R v3.5.2 [41]. The mean read-depth across the pseudomolecules was largely consistent with the scaffold sex-assignment i.e. zero depth for males and haploid for females for the W chromosome (Figure 4C) and neoW-1A (Figure 4A), diploid depth for males and haploid for females for the Z chromosome (Figure 4D) and neoZ-1A (Figure 4B), and diploid depth for both sexes for autosomal chromosome 5 (Fig. 4E). In contrast to the W and Z chromosomes, several distinct genomic regions with read-coverage consistent with that of an autosomal chromosome (Fig 4E) were observed for neoW-1A (Figure 4A) and neoZ-1A (Figure 4B), mostly at the pseudomolecule termini.

Identification of chromosome 1A-anchored gametologous gene pairs

Using FastANI, we calculated the pairwise sequence identity between the neoW-1A or neoZ-1A pseudomolecule and the Zebra Finch chromosome 1A [42] and found that both exhibited substantial sequence identity (calculated mean nucleotide identify of 86%) across the whole of Zebra Finch chromosome 1A (Figure 5A, B). NeoW-1A exhibited ~ 20 Mb greater assembled length (92.5 Mb) than did neoZ-1A (72.5 Mb) (Figure 5 A, B). Accumulation of repeats contributed to this: 36.6% of the EYR neoW-1A sequence was characterized as repetitive by RepeatMasker, while this value is only 10% for the EYR neoZ-1A sequence. Also using FastANI, we calculated the pairwise sequence identity between EYR neoW-1A and neoZ-1A in a non-overlapping sliding window of 10 kb. By aligning the putative neoW-1A to the neoZ-A, we observed high (mostly >90%) pairwise sequence identity throughout the pseudomolecule (Figure 5C). However, there was considerable heterogeneity in absolute sequence similarity, with zones of ~100 %, ~98 %, ~95 %, and ~92 % identity clumped along the pseudomolecules, suggestive of evolutionary strata (Figure 5C) [43].

Orthologous genes shared between the EYR and Collared Flycatcher (higher protein similarity to EYR compared to Zebra Finch) were inferred using Orthofinder2 [44]. Of the 957 genes located on the Collared Flycatcher chromosome 1A, 725 formed a one-to-one (N=513) or one-to-many (N=212) orthologous group with the EYR genes located on the neoZ-1A or neoW-1A pseudomolecule. We restricted the ortholog analysis to only genes predicted from the sex-linked scaffolds (identified based on EYR scaffold assignment) since the “autosomal-behaving” scaffolds on the neoZ-1A and neoW-1A pseudomolecules (Figure 4A and B) may consist of unassigned sex-linked, recombining sex-linked (collapsed into a single scaffold) or truly autosomal scaffolds that will affect gametologous pairing. This resulted in the identification of 419 Z-linked genes on EYR neoZ-1A pseudomolecule and 221 W-linked orthologs on neoW-1A, to a total of 488 different sex-linked genes. Among these were 148 putative gametologous gene pairs (i.e. homologs with sufficiently low recombination for one version to be identifiably W- and one Z-linked) between EYR neoW- and neoZ-1A (Supplemental Table 2). The fewer number of W-linked EYR genes that formed orthologous group with the Collared Flycatcher chromosome 1A genes compared to that of Z-linked EYR genes may be due to the more fragmented assembly and higher repetitiveness of W-linked EYR scaffolds that precludes the accurate annotation of genes in the W-linked scaffolds when using the default BRAKER2 annotation settings [45]. It is also possible that W-linked EYR genes on chromosome 1A have diverged, been lost or degraded beyond detection, as expected under sex chromosome evolution [43].

Neo-sex chromosomes have reduced effective population size relative to the autosomes that contribute to them: this is expected to decreased the effectiveness of purifying selection, especially when compounded by reduced recombination [46]. These effects should promote the accumulation of deleterious mutations, commonly revealed as elevated non-synonymous to synonymous (dN/dS) ratios, particularly for sex-limited chromosomes such as the neo-W in birds [7, 8, 47]. To calculate the dN/dS ratios for EYR neo-sex gametologous gene pairs, protein alignment was first performed for all 148 putative neo-sex gametologous gene pairs with their respective Collared Flycatcher orthologs using Clustal Omega v1.2.1 [48] followed by codon-based alignment with pal2nal (-nogap option to remove gaps and inframe stop codons) [49]. The pal2nal output for each orthologous group was used to calculate dN/dS ratios via codeml in paml v4.9i package [50]. When the Collared Flycatcher orthologous 1A genes were used as the reference for each comparison, 120 of 148 neoW-1A gametologs exhibited higher dN/dS than their neoZ-1A gametologous partners (Wilcoxon paired samples signed rank test, $p = 3.9e-14$; Fig. 5 D).

Conclusion

We report a hybrid genome assembly using Nanopore and Illumina reads of a female Eastern Yellow Robin (EYR), the first published genome for the family Petroicidae. The identification of sex-linked scaffolds using a combination of read-depth and *k*-mer YGS approaches followed by chromosomal anchoring to the genome of a female Zebra Finch, provided strong evidence for the presence of a neo-sex chromosome system in EYR involving most of chromosome 1A. The inferred neoW-1A pseudomolecule showed the characteristics expected of a sex-limited neo-sex chromosome, including elevated dN/dS ratios, increased levels of repetitive sequences and signals of strata of levels of sequence divergence [7, 8, 47]. Further work is required to understand the formation of the neo-sex system we infer. One relatively simple model is that one copy of chromosome 1A fused with the W chromosome, and the second copy of chromosome 1A became inherited in a neo-Z fashion; but more complicated scenarios are possible [7, 8, 15]. Given that the divergence between inland and coastal EYR lineages is partly due to a genomic region enriched for nuclear genes with mitochondrial functions that maps to the autosomal chromosome 1A in other songbirds [3](Morales et al. 2018), the role of neo-sex chromosomes in maintaining lineage divergence despite nuclear gene flow warrants further investigation involving a female genome of the coastal lineage. Future work should also test whether unlikely but possible neoZ-1A difference between the EYR054 used for assembly here and EYR056 used for scaffolding affected the neo-Z assembly. Chromosome 1A is not one of the chromosomes implicated in multiple known vertebrate neo-sex systems [47], but given its unusual concentration of genes with mitochondrial functions, it will not be surprising if subsequent equivalent cases are found [3]. Our results show that assuming close synteny between a songbird of interest and a distantly-related reference genome can lead to incomplete or incorrect evolutionary inferences. The present genome assembly will be an important molecular resource for understanding and re-evaluating genome evolution in EYR, a key model wildlife species in the emerging field of ‘mitonuclear ecology’ [4]. The discovery of neo-sex chromosomes in this bird species adds another, independent model to the limited number in which sex chromosome evolution can be studied through the lens of relatively young sex chromosomes [15, 47].

Availability of supporting data

The genome assembly has been deposited in the NCBI database with the accession number QKXG010000000. Raw sequencing data have been deposited in the NCBI Sequence Read Archive (SRA) database and linked to the Bioproject ID PRJNA476023.

Competing interests

The authors declare that they have no competing interests.

Funding

This study was funded by the Monash School of Biological Sciences, Australian Research Council (ARC) Discovery Project (DP180102359), ARC Linkage Grant (LP0776322), the Victorian Department of Sustainability and Environment (DSE), Museum of Victoria, Victorian Department of Primary Industries, Parks Victoria, North Central Catchment Management Authority, Goulburn Broken Catchment Management Authority, CSIRO Ecosystem Sciences, and the Australian National Wildlife Collection Foundation.

Authors’ contributions

HMG, PS and AP conceived the study. HEM and SF collected the samples and extracted the genomic DNA. CMA contributed sequencing reagents and computing resources. HMG performed whole-genome sequencing, genome assembly, genome annotation and comparative genomics analysis. HEM assessed the assembly quality and genomics analysis. HMG, AP and PS wrote the manuscript. All authors read, edited and approved the final manuscript.

Acknowledgments

We thank Nevil Amos, Lana Austin and all other collectors of specimens and all agencies who granted permission to collect specimens. Field samples were collected under scientific research permits issued by the Victorian Department of Environment and Primary Industries (numbers 10007165, 10005919 and 10005514), New South Wales Office of Environment and Heritage (SL100886), in accordance with Animal Ethics approvals AM13-05, BSCI_2012_20 and BSCI_2007_07, using bands issued by the Australian Bird and Bat Banding Scheme. We also thank the Australian National Wildlife Collection, Leo Joseph, Robert Palmer and Richard Major for providing genetic samples. We are grateful to Erich Jarvis and Olivier Fedrigo for giving us access to the female Zebra Finch assembly. We thank Gabriel Low for helpful discussion.

Figure legends

Figure 1. The Eastern Yellow Robin. Photo by Geoff Park

Figure 2. Genomic profiling and *in-silico* sexing of Eastern Yellow Robin. (A) Genomescope profile calculated from trimmed Illumina data of EYR054 using a *k*-mer length of 25. (B) Median coverage per individual for three sets of scaffolds with different inheritance, for the female sample (N=10) and male sample (N=9), with individuals sequenced at ~ 10x coverage each. Autosomal = GAPDH-containing chromosome fragment [1] scaffold QKXG0002030; W = W-chromosome fragment: scaffold QKXG0001703; Z = Z chromosome fragment: scaffold QKXG0001459. (C) Frequency distribution of base-by-base read-depth calculated from the mapping of pooled male (blue line) and female (red line) reads to the female genome assembly. This is subsequently used to estimate the read-depth of haploid and diploid scaffolds.

Figure 3. The assembled lengths of Eastern Yellow Robin (EYR) chromosome 1A, W and Z pseudomolecules constructed by anchoring different scaffold inputs to the female Zebra Finch reference genome (ZF; grey bars). Inputs included: EYR genome (EYR; yellow bars); EYR candidate W-linked scaffolds (EYR W-linked; red bars); EYR candidate Z-linked scaffolds (EYR Z-linked; dark blue bars); EYR genome without Z-linked scaffolds (EYR w/o Z-linked; pink bars); EYR genome without W-linked scaffolds (EYR w/o W-linked; light blue bars). Neo-sex-chromosome pseudomolecules were built using the latter two datasets (the length of neoW-1A is shown by the light blue bar and that of neoZ-1A by the light pink bar for Ch1A).

Figure 4. Read-depth of pooled male (blue dots) and pooled female (red dots) reads across EYR pseudomolecules neoW-1A, neoZ-1A, W, Z and autosomal chromosome 5. Read-depth (the number of reads for each nucleotide in the genome) was estimated for each 100 kb sliding window. The locations of candidate W-linked scaffolds on the neoW-1A and Z-linked scaffolds on neoZ-1A pseudomolecules are indicated by the black lines below the read-depth plots. Coordinate (Mb) refers to the position on the pseudomolecule.

Figure 5. Characterization of the inferred neo-sex chromosomes in Eastern Yellow Robin. Linear genome comparison of the (A) neoW-1A and (B) neoZ-1A pseudomolecules (blue horizontal bars) with the Zebra Finch chromosome 1A (green horizontal bars). The neoW-1A alignment is ~ 20 Mb longer than that of neoZ-1A. The red lines denote regions of nucleotide similarity with more than

70% nucleotide identity calculated over 10 kb non-overlapping sliding window. (C) Pairwise sequence identity per 10 kb sliding window (to obtain high resolution) between the neoW-1A and neoZ-1A scaffolds mapped along the neoZ-1A pseudomolecule, with coordinates relating to the neoZ-1A pseudomolecule. Zones of different levels of sequence similarity can be seen along the pseudomolecule. The blue line denotes the smoothed conditional means for pairwise identity and the grey zone around it indicates the 95% confidence interval. (D) Paired box plots showing the dN/dS ratios of neoW-1A and neoZ-1A gene copies (gametologs) of the Eastern Yellow Robin compared with Collared Flycatcher orthologs as references. Collared Flycatcher was used here in preference to Zebra Finch because the former has greater protein similarity to EYR. Grey lines connecting red and blue dots represent gametologs.

Table 1

Table 1. Genome assembly and annotation statistics of the Eastern Yellow Robin

Parameter	Details
Organism	*Eopsaltria australis* (Eastern Yellow Robin)
Isolate	EYR054 (sex = female; data type: Illumina standard paired-end and Nanopore long read)
	EYR056 (sex = female; data type: Illumina mate-pair)
Bioproject	PRJNA476023
Biosample	SAMN09425179 (isolate EYR054)
	SAMN10581952 (isolate EYR056)
GenBank assembly accession	GCA_003426825.1 (QKXG01)
Assembled Length	1,228,344,903 bp
Scaffold N_{50}	987,278 bp
Number of scaffolds	20,702
Number of predicted genes	23,905

**Repeat Annotation:**	
LINEs	39,888,415 bp (3.25%)
LTR elements	85,519,635 (6.96%)
DNA elements	6,416,492 (0.52%)
Unclassified repeats	42,749,317 (3.48%)
Satellites	1,967,923 (0.16%)
Simple repeats	14,300,770 (1.16%)
Low complexity	3,128,912 (0.25%)

**BUSCO completeness (Ave odb9)**	Whole genome	Predicted proteome
Complete BUSCO	4,627 (94.2%)	3,795 (77.2%)
Complete and single-copy BUSCO	4,436 (90.3%)	3,302 (67.2%)
Complete and duplicated BUSCO	191 (3.9%)	493 (10.0%)
Fragmented BUSCO	163 (3.3%)	590 (12.0%)
Missing BUSCO	125 (2.5%)	530 (10.8%)
Total BUSCO groups search	4,915	4,915

References

1. Pavlova A, Amos JN, Joseph L, Loynes K, Austin JJ, Keogh JS, et al. Perched at the mito-nuclear crossroads: divergent mitochondrial lineages correlate with environment in the face of ongoing nuclear gene flow in an Australian bird. Evolution. 2013;67 12:3412-28.
2. Morales HE, Sunnucks P, Joseph L and Pavlova A. Perpendicular axes of differentiation generated by mitochondrial introgression. Molecular ecology. 2017;26 12:3241-55.
3. Morales HE, Pavlova A, Amos N, Major R, Kilian A, Greening C, et al. Concordant divergence of mitogenomes and a mitonuclear gene cluster in bird lineages inhabiting different climates. Nature ecology & evolution. 2018;2 8:1258.
4. Hill GE. Mitonuclear ecology. Oxford, United Kingdom: Oxford University Press; 2019.
5. Warren WC, Clayton DF, Ellegren H, Arnold AP, Hillier LW, Künstner A, et al. The genome of a songbird. Nature. 2010;464:757. doi:10.1038/nature08819

https://www.nature.com/articles/nature08819#supplementary-information.

6. Sunnucks P, Morales HE, Lamb AM, Pavlova A and Greening C. Integrative approaches for studying mitochondrial and nuclear genome co-evolution in oxidative phosphorylation. Frontiers in genetics. 2017;8:25.
7. Dierickx E, Sin S, van Veelen P, Brooke MdL, Liu Y, Edwards S, et al. Neo-sex chromosomes and demography shape genetic diversity in the Critically Endangered Raso lark. bioRxiv. 2019:617563. doi:10.1101/617563.
8. Leroy T, Anselmetti Y, Tilak M-K, Bérard S, Csukonyi L, Gabrielli M, et al. A bird's white-eye view on neosex chromosome evolution. bioRxiv. 2019:505610. doi:10.1101/505610.
9. Austin CM, Tan MH, Harrisson KA, Lee YP, Croft LJ, Sunnucks P, et al. De novo genome assembly and annotation of Australia's largest freshwater fish, the Murray cod (Maccullochella peelii), from Illumina and Nanopore sequencing read. Gigascience. 2017;6 8:1-6.
10. Tan MH, Austin CM, Hammer MP, Lee YP, Croft LJ and Gan HM. Finding Nemo: hybrid assembly with Oxford Nanopore and Illumina reads greatly improves the clownfish (Amphiprion ocellaris) genome assembly. GigaScience. 2018;7 3:gix137.
11. Zimin AV, Puiu D, Luo M-C, Zhu T, Koren S, Marçais G, et al. Hybrid assembly of the large and highly repetitive genome of *Aegilops tauschii*, a progenitor of bread wheat, with the MaSuRCA mega-reads algorithm. Genome research. 2017.
12. Zimin AV, Puiu D, Hall R, Kingan S, Clavijo BJ and Salzberg SL. The first near-complete assembly of the hexaploid bread wheat genome, Triticum aestivum. Gigascience. 2017;6 11:1-7. doi:10.1093/gigascience/gix097.
13. Morales HE, Pavlova A, Joseph L and Sunnucks P. Positive and purifying selection in mitochondrial genomes of a bird with mitonuclear discordance. Mol Ecol. 2015;24 11:2820-37. doi:10.1111/mec.13203.
14. Sardell J. *Evolutionary Consequences of Recent Secondary Contact Between Myzomela Honeyeaters*. 2016.
15. Pala I, Naurin S, Stervander M, Hasselquist D, Bensch S and Hansson B. Evidence of a neo-sex chromosome in birds. Heredity (Edinb). 2012;108 3:264-72. doi:10.1038/hdy.2011.70.
16. Brooke Mde L, Welbergen JA, Mainwaring MC, van der Velde M, Harts AM, Komdeur J, et al. Widespread translocation from autosomes to sex chromosomes preserves genetic variability in an endangered lark. Journal of molecular evolution. 2010;70 3:242-6. doi:10.1007/s00239-010-9333-3.
17. Harrisson KA, Pavlova A, Amos JN, Takeuchi N, Lill A, Radford JQ, et al. Fine-scale effects of habitat loss and fragmentation despite large-scale gene flow for some regionally declining woodland bird species. Landscape Ecology. 2012;27 6:813-27. doi:10.1007/s10980-012-9743-2.
18. Morales HE, Pavlova A, Sunnucks P, Major R, Amos N, Joseph L, et al. Neutral and selective drivers of colour evolution in a widespread Australian passerine. Journal of biogeography. 2017;44 3:522-36.
19. Li H. Minimap2: pairwise alignment for nucleotide sequences. Bioinformatics. 2018;34 18:3094-100. doi:10.1093/bioinformatics/bty191.
20. Amos JN, Harrisson KA, Radford JQ, White M, Newell G, Nally RM, et al. Species- and sex-specific connectivity effects of habitat fragmentation in a suite of woodland birds. Ecology. 2014;95 6:1556-68. doi:10.1890/13-1328.1.
21. Griffiths R, Double MC, Orr K and Dawson RJ. A DNA test to sex most birds. Molecular ecology. 1998;7 8:1071-5.
22. Chen S, Zhou Y, Chen Y and Gu J. fastp: an ultra-fast all-in-one FASTQ preprocessor. Bioinformatics. 2018;34 17:i884-i90. doi:10.1093/bioinformatics/bty560.
23. Marçais G and Kingsford C. A fast, lock-free approach for efficient parallel counting of occurrences of k-mers. Bioinformatics. 2011;27 6:764-70. doi:10.1093/bioinformatics/btr011.
24. Vurture GW, Sedlazeck FJ, Nattestad M, Underwood CJ, Fang H, Gurtowski J, et al. GenomeScope: fast reference-free genome profiling from short reads. Bioinformatics. 2017;33 14:2202-4.

25. Zimin AV, Marçais G, Puiu D, Roberts M, Salzberg SL and Yorke JA. The MaSuRCA genome assembler. Bioinformatics. 2013;29 21:2669-77.
26. Paulino D, Warren RL, Vandervalk BP, Raymond A, Jackman SD and Birol I. Sealer: a scalable gap-closing application for finishing draft genomes. BMC bioinformatics. 2015;16 1:230.
27. Sahlin K, Chikhi R and Arvestad L. Assembly scaffolding with PE-contaminated mate-pair libraries. Bioinformatics. 2016;32 13:1925-32.
28. Waterhouse RM, Seppey M, Simão FA, Manni M, Ioannidis P, Klioutchnikov G, et al. BUSCO applications from quality assessments to gene prediction and phylogenomics. Molecular biology and evolution. 2017;35 3:543-8.
29. Smit AF, Hubley R and Green P. RepeatMasker. 1996.
30. Smit A and Hubley R. RepeatModeler Open-1.0. Available fom http://www repeatmasker org. 2008.
31. Ellegren H, Smeds L, Burri R, Olason PI, Backström N, Kawakami T, et al. The genomic landscape of species divergence in Ficedula flycatchers. Nature. 2012;491 7426:756.
32. Hoff KJ, Lange S, Lomsadze A, Borodovsky M and Stanke M. BRAKER1: unsupervised RNA-Seq-based genome annotation with GeneMark-ET and AUGUSTUS. Bioinformatics. 2015;32 5:767-9.
33. Langmead B and Salzberg SL. Fast gapped-read alignment with Bowtie 2. Nature methods. 2012;9 4:357.
34. Lindenbaum P. JVarkit: java-based utilities for Bioinformatics. 2015.
35. Lindenbaum P and Redon R. bioalcidae, samjs and vcffilterjs: object-oriented formatters and filters for bioinformatics files. Bioinformatics. 2018;34 7:1224-5. doi:10.1093/bioinformatics/btx734.
36. Li H, Handsaker B, Wysoker A, Fennell T, Ruan J, Homer N, et al. The Sequence Alignment/Map format and SAMtools. Bioinformatics. 2009;25 16:2078-9. doi:10.1093/bioinformatics/btp352.
37. Carvalho AB and Clark AG. Efficient identification of Y chromosome sequences in the human and Drosophila genomes. Genome research. 2013;23 11:1894-907. doi:10.1101/gr.156034.113.
38. Xu L, Auer G, Peona V, Suh A, Deng Y, Feng S, et al. Dynamic evolutionary history and gene content of sex chromosomes across diverse songbirds. Nat Ecol Evol. 2019;3 5:834-44. doi:10.1038/s41559-019-0850-1.
39. Alonge M, Soyk S, Ramakrishnan S, Wang X, Goodwin S, Sedlazeck FJ, et al. Fast and accurate reference-guided scaffolding of draft genomes. bioRxiv. 2019:519637. doi:10.1101/519637.
40. Quinlan AR and Hall IM. BEDTools: a flexible suite of utilities for comparing genomic features. Bioinformatics. 2010;26 6:841-2. doi:10.1093/bioinformatics/btq033.
41. Wickham H. ggplot2: Elegant Graphics for Data Analysis. Springer Publishing Company, Incorporated; 2009.
42. Jain C, Rodriguez-R LM, Phillippy AM, Konstantinidis KT and Aluru S. High throughput ANI analysis of 90K prokaryotic genomes reveals clear species boundaries. Nature communications. 2018;9 1:5114.
43. Wright AE, Dean R, Zimmer F and Mank JE. How to make a sex chromosome. Nature communications. 2016;7:12087.
44. Emms DM and Kelly S. OrthoFinder: solving fundamental biases in whole genome comparisons dramatically improves orthogroup inference accuracy. Genome biology. 2015;16 1:157.
45. Rutkowska J, Lagisz M and Nakagawa S. The long and the short of avian W chromosomes: no evidence for gradual W shortening. Biology letters. 2012;8 4:636-8. doi:10.1098/rsbl.2012.0083.
46. Smeds L, Warmuth V, Bolivar P, Uebbing S, Burri R, Suh A, et al. Evolutionary analysis of the female-specific avian W chromosome. Nature communications. 2015;6:7330.
47. Sigeman H, Ponnikas S, Videvall E, Zhang H, Chauhan P, Naurin S, et al. Insights into Avian Incomplete Dosage Compensation: Sex-Biased Gene Expression Coevolves with Sex Chromosome Degeneration in the Common Whitethroat. Genes. 2018;9 8 doi:10.3390/genes9080373.
48. Sievers F and Higgins DG. Clustal Omega, accurate alignment of very large numbers of sequences. Multiple sequence alignment methods. Springer; 2014. p. 105-16.

49. Suyama M, Torrents D and Bork P. PAL2NAL: robust conversion of protein sequence alignments into the corresponding codon alignments. Nucleic acids research. 2006;34 suppl_2:W609-W12.
50. Yang Z. PAML 4: phylogenetic analysis by maximum likelihood. Molecular biology and evolution. 2007;24 8:1586-91.

Figure1

Click here to download Figure Fig1_birdpic.jpg

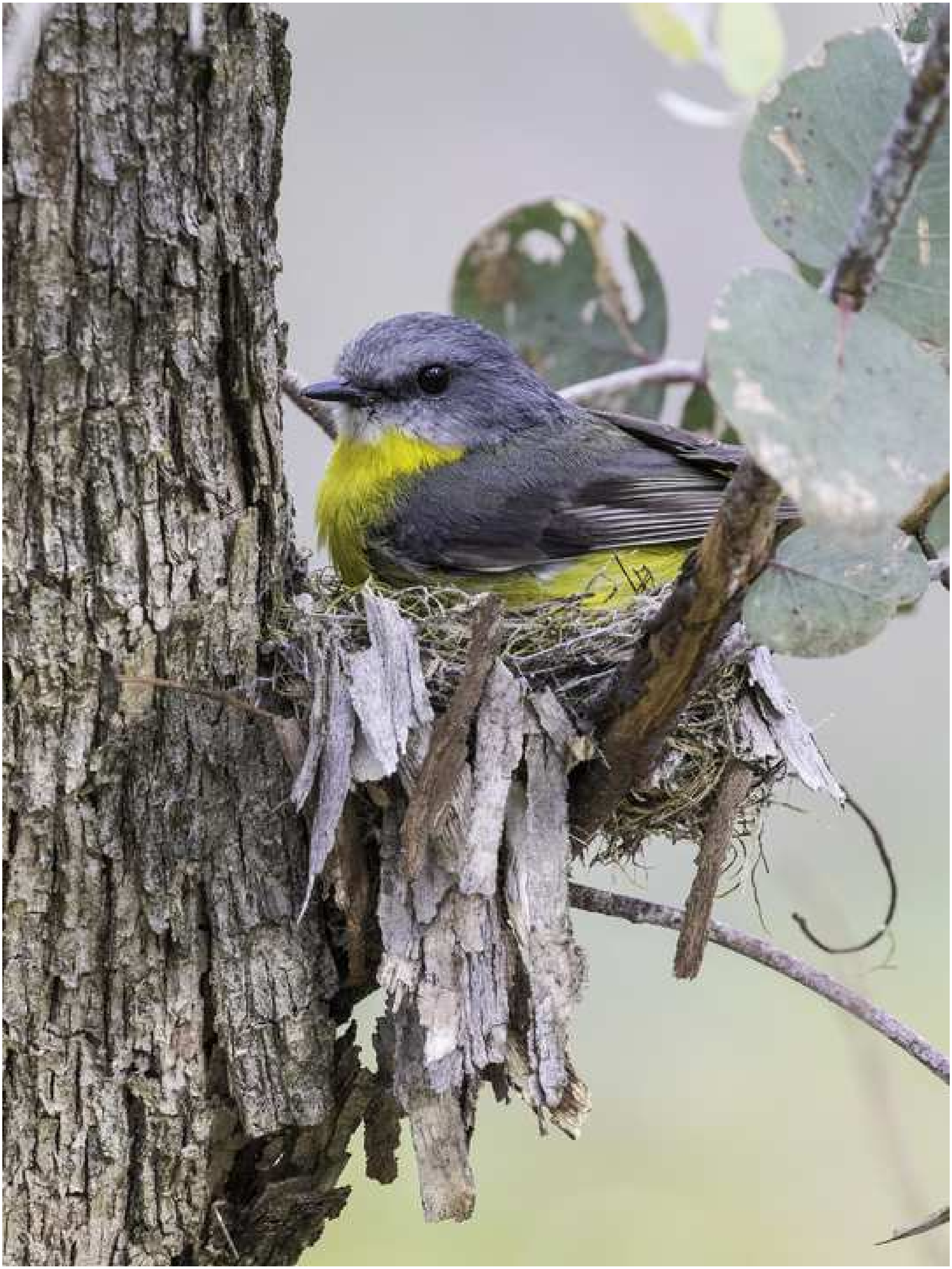

Figure2

Click here to download Figure Fig2_generalstats.tif

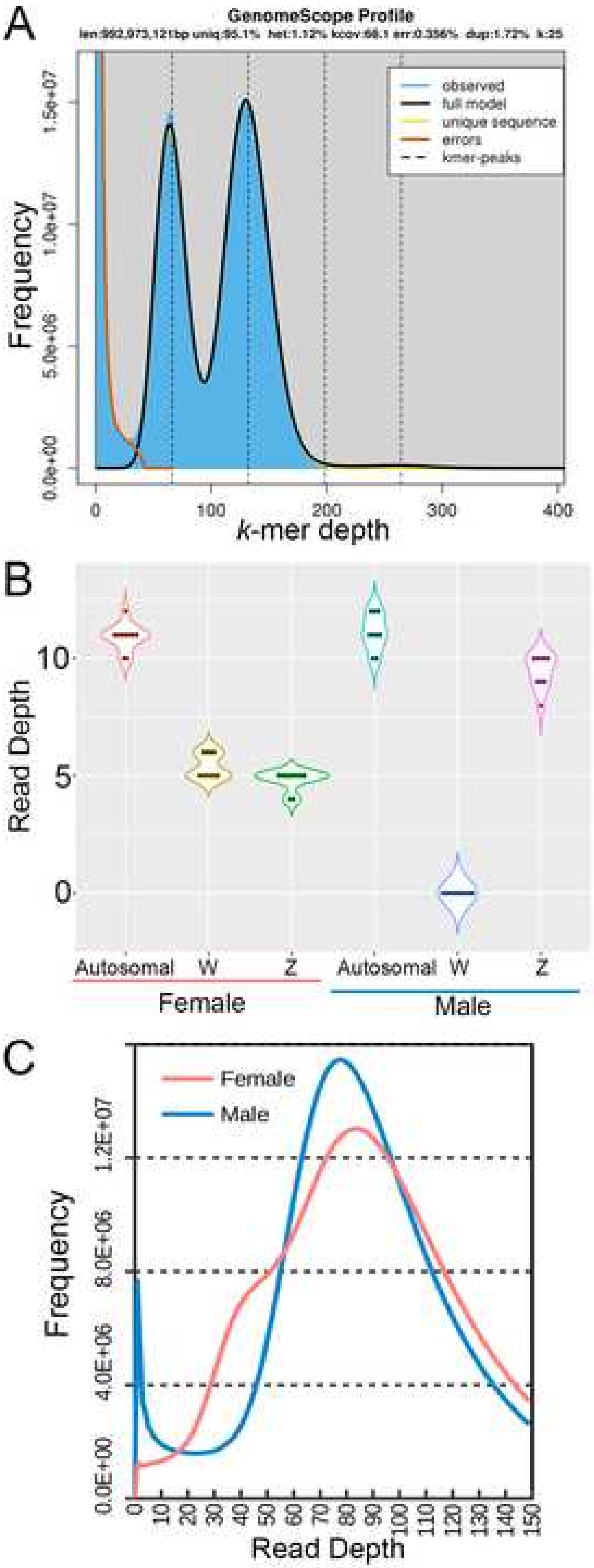

Figure3

Click here to download Figure Fig3_ragoo.tif

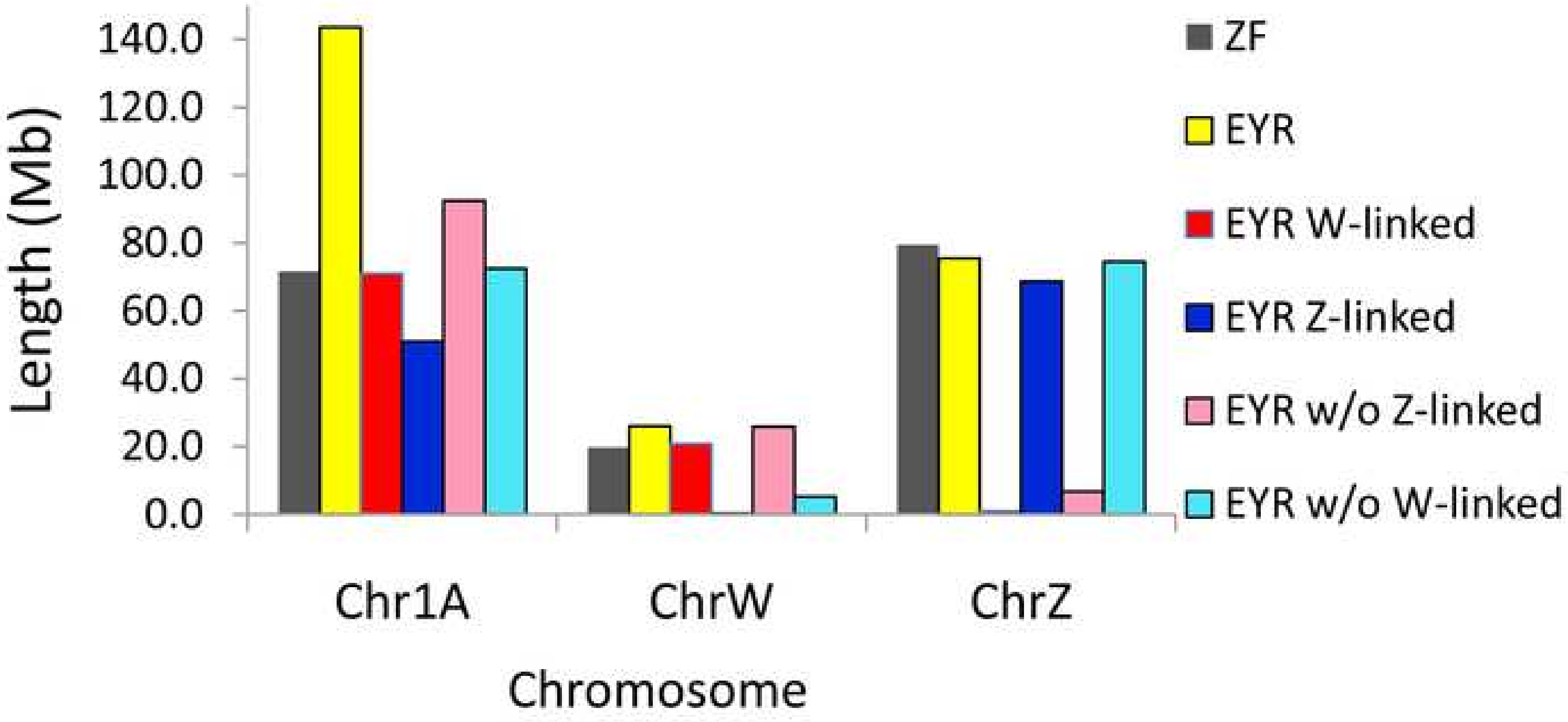

Click here to download Figure Fig4_cov.tif

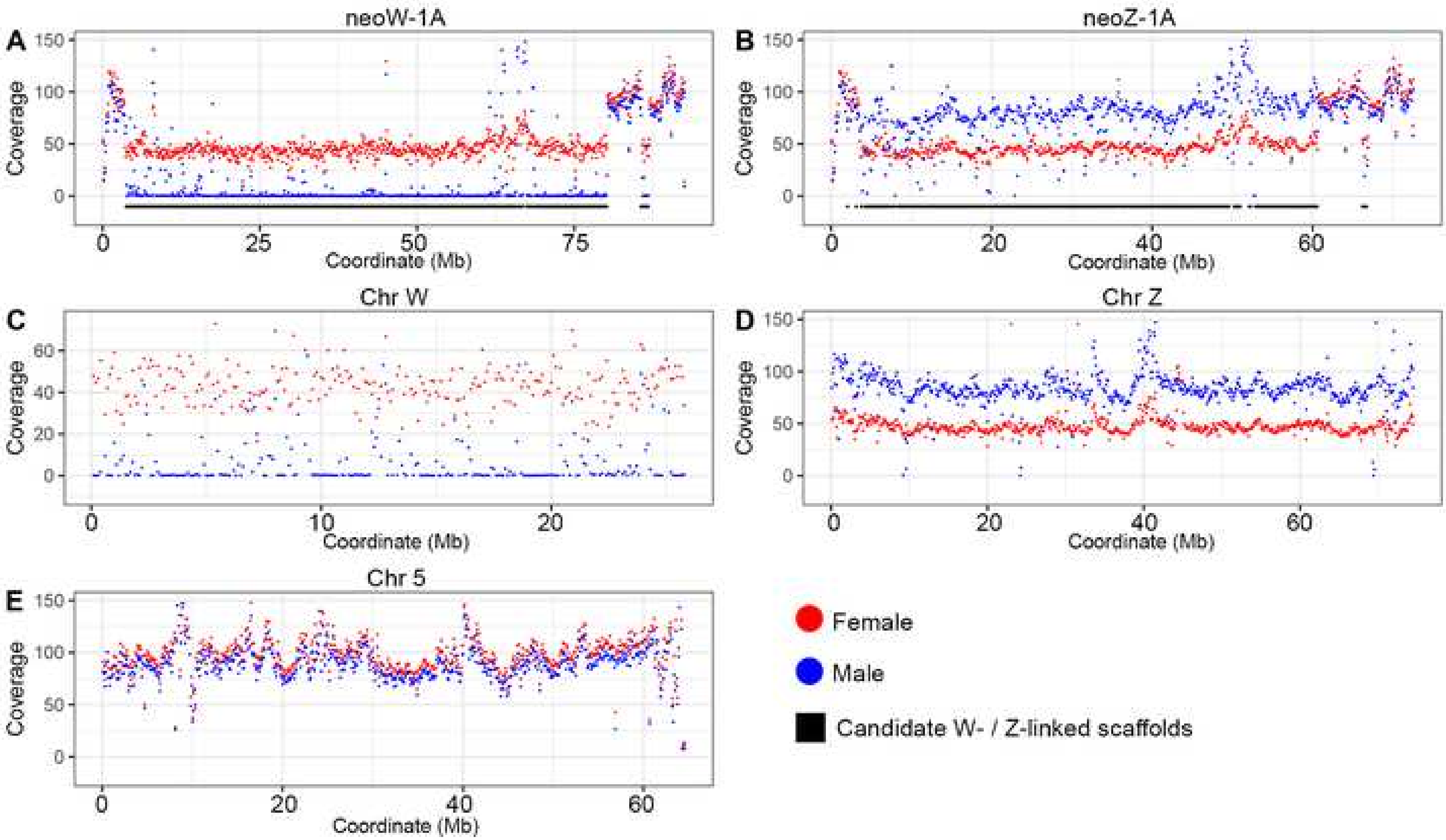

Figure5

Click here to download Figure Fig5_dnds.tif

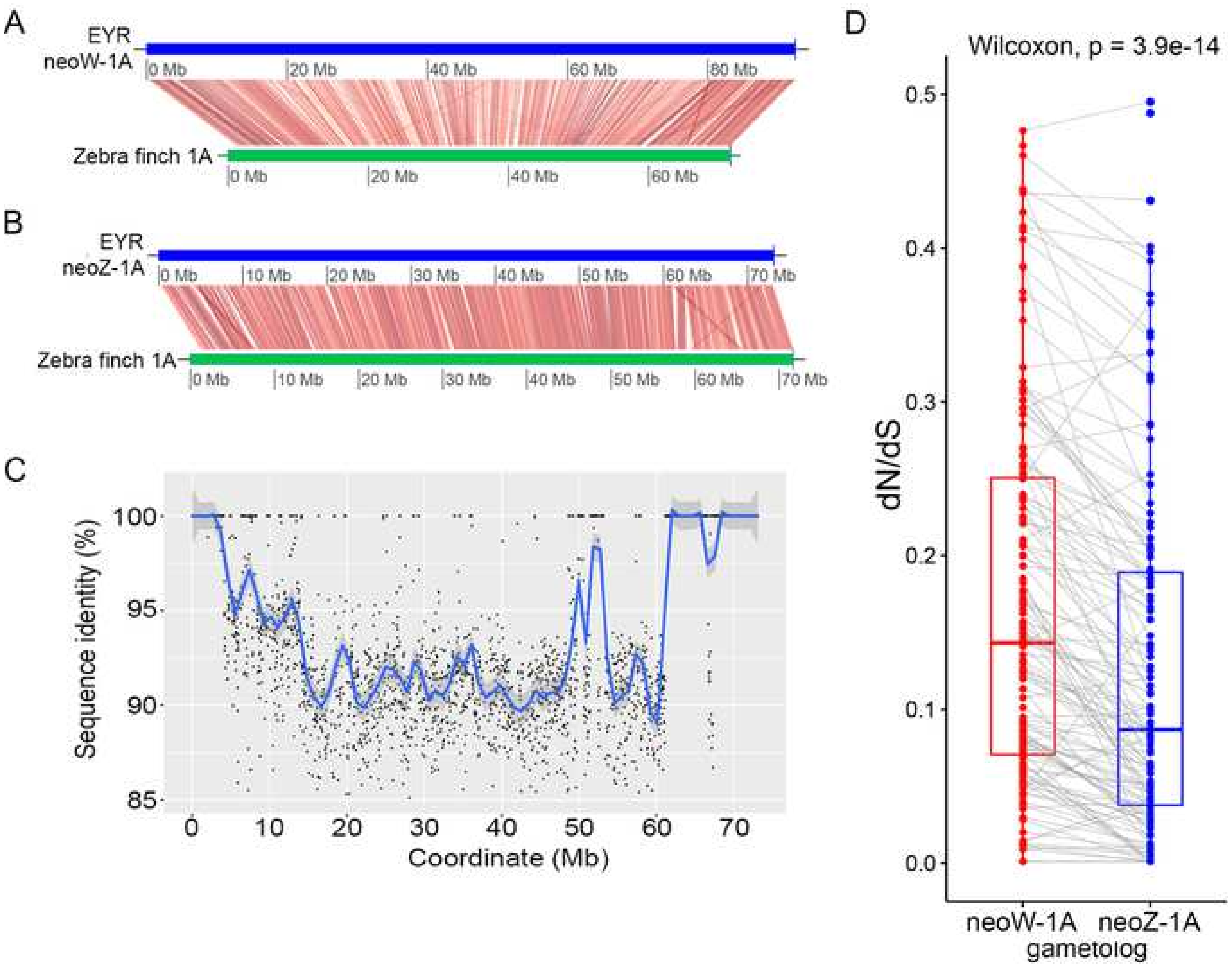

Click here to access/download

Supplementary Material

EYR_Response_250619.docx

Supplementary Table 1

Click here to access/download
Supplementary Material
SupplementalTable1.xlsx

Supplementary Table 2

Click here to access/download
Supplementary Material
SupplementalTable2.xls
