# Supplementary material for: Genomic evidence of neo-sex chromosomes in the eastern yellow robin
Source: Gigascience. 2019 Sep 3;8(9):giz111. doi: 10.1093/gigascience/giz111 (PMC6736294; doi:10.1093/gigascience/giz111)
Supplement: giz111_Response_to_Reviewer_Comments_Original_Submission [file giz111_response_to_reviewer_comments_original_submission.pdf]

Author's Response To Reviewer Comments

Close

Editor:
-In your revised manuscript, please include a citation to your upcoming GigaDB dataset (including the DOI link) to your reference list, and cite this in the data availability section and elsewhere in the manuscript, where appropriate.
-In the data availability section, please write something along the lines of " Supporting data, including the genome assembly, annotations, and [... mention other data types...] are available via the GigaScience database, GigaDB [xx]."

Added GigaDB in the data availability citation as per recommended format. (DOI yet to be made available)

Reviewer #1:

-I did not understand the reasoning behind the "evolutionary strata" argument in Figure 5C. I only realized what the point of this analysis was when I read the suggestion by Reviewer 3. Please explain this better in the ms.

We have adjusted the text accordingly:
One expectation under sex chromosome evolution is the development of evolutionary strata – regions of suppressed recombination identified by spatial clusters of Z-W orthologs with similar divergence estimates [43].

-Line 54: "sex-linked genome region". genomic or regions.
Genome region -> genomic regions

-line 174: Remove underlined ".".
Removed

-line 264: "Surprisingly,A substantial". Fix capital "A"..
Fixed

Reviewer #2:

Line 264 - "A" should be lowercase
Fixed

Line 326 - Fix "…to decrease…"
Fixed

Line 327 - More appropriate citations would be Charlesworth & Charlesworth 2000 Phil Trans Roy Soc B

and Charlesworth 1994 Genet Res. Also, note that reduced purifying selection due to the reduced effective population sizes of the W/Y chromosomes is only one of the reasons why we expect accumulation of deleterious mutations, along with Mueller's ratchet and hitchhiking (see Bachtrog 2006 Current Opinion in Genetics & Development).

Citations added. We also added ‘along with Mueller's ratchet and hitchhiking’.

Line 345 - Remove comma (or insert comma between "approaches" and "followed" in previous line)
Inserted comma in previous line.

Line 357 - Fix to "neoZ-1A differences"
Fixed

Figure 2A - This figure is not currently cited in the manuscript

Did a search in Microsoft word and confirm that Figure 2A is cited in the manuscript.

“…..genome profiling based on Jellyfish2-calculated k-mer frequency (k=25) that estimated a genome size of 993 Mb with 1.12% heterozygosity for EYR054 (Figure 2A) [22-24]. We used MaSuRCA v3.2.4….”

Close
