# Supplementary material for: Genomic evidence of neo-sex chromosomes in the eastern yellow robin
Source: Gigascience. 2019 Sep 3;8(9):giz111. doi: 10.1093/gigascience/giz111 (PMC6736294; doi:10.1093/gigascience/giz111)
Supplement: giz111_Reviewer_2_Report_Original_Submission — Jason Sardell -- 7/29/2019 Reviewed [file giz111_reviewer_2_report_original_submission.pdf]

Reviewer Report

Title: Genomic evidence of neo-sex chromosomes in the Eastern Yellow Robin

Version: Original Submission **Date:** 7/29/2019

Reviewer name: Jason Sardell

Reviewer Comments to Author:

The current version of the manuscript by Gan et al. is substantially improved over the previous version. The context of their study is better developed, and they make a strong case in the abstract and introduction for the value of an EYR reference genome. They also provide clear and convincing evidence for a neo-sex chromosome in this species. Notably, scaffolds that map to chr1A in the Zebra Finch reference genome, which is not known to be sex-linked in any other species of bird, are sex-linked in EYR. The methods that they apply to demonstrate this surprising and noteworthy finding are appropriate and consistent with those employed by many other recent papers investigating similar systems. I recommend that their manuscript be accepted for publication in GigaScience and look forward to their future work with this interesting system.
Sincerely,
Jason M. Sardell
Minor corrections:
Line 264 - "A" should be lowercase
Line 326 - Fix "…to decrease…"
Line 327 - More appropriate citations would be Charlesworth & Charlesworth 2000 Phil Trans Roy Soc B and Charlesworth 1994 Genet Res. Also, note that reduced purifying selection due to the reduced effective population sizes of the W/Y chromosomes is only one of the reasons why we expect accumulation of deleterious mutations, along with Mueller's ratchet and hitchhiking (see Bachtrog 2006 Current Opinion in Genetics & Development).
Line 345 - Remove comma (or insert comma between "approaches" and "followed" in previous line)
Line 357 - Fix to "neoZ-1A differences"
Figure 2A - This figure is not currently cited in the manuscript

Level of Interest

Please indicate how interesting you found the manuscript: Choose an item.

Quality of Written English

Please indicate the quality of language in the manuscript: Choose an item.

Declaration of Competing Interests

Please complete a declaration of competing interests, considering the following questions:

- Have you in the past five years received reimbursements, fees, funding, or salary from an organisation that may in any way gain or lose financially from the publication of this manuscript, either now or in the future?
- Do you hold any stocks or shares in an organisation that may in any way gain or lose financially from the publication of this manuscript, either now or in the future?
- Do you hold or are you currently applying for any patents relating to the content of the manuscript?
- Have you received reimbursements, fees, funding, or salary from an organization that holds or has applied for patents relating to the content of the manuscript?
- Do you have any other financial competing interests?
- Do you have any non-financial competing interests in relation to this paper?

If you can answer no to all of the above, write 'I declare that I have no competing interests' below. If your reply is yes to any, please give details below.

I declare that I have no competing interests

I agree to the open peer review policy of the journal. I understand that my name will be included on my report to the authors and, if the manuscript is accepted for publication, my named report including any attachments I upload will be posted on the website along with the authors' responses. I agree for my report to be made available under an Open Access Creative Commons CC-BY license (http://creativecommons.org/licenses/by/4.0/). I understand that any comments which I do not wish to be included in my named report can be included as confidential comments to the editors, which will not be published.

Choose an item.

To further support our reviewers, we have joined with Publons, where you can gain additional credit to further highlight your hard work (see: https://publons.com/journal/530/gigascience). On publication of this paper, your review will be automatically added to Publons, you can then choose whether or not to claim your Publons credit. I understand this statement.

Yes Choose an item.
